# A *C1173T* Dimorphism in the *VKORC1* Gene Determines Coumarin Sensitivity and Bleeding Risk

**DOI:** 10.1371/journal.pmed.0020312

**Published:** 2005-10-11

**Authors:** Pieter H Reitsma, Jeroen F. van der Heijden, Angelique P Groot, Frits R Rosendaal, Harry R Büller

**Affiliations:** **1**Laboratory for Experimental Internal Medicine, Academic Medical Center, University of Amsterdam, Amsterdam, The Netherlands,; **2**Departments of Clinical Epidemiology and Hematology, Leiden University Medical Center, Leiden, The Netherlands,; **3**Department of Vascular Medicine, Academic Medical Center, University of Amsterdam, Amsterdam, The Netherlands; University of AberdeenUnited Kingdom

## Abstract

**Background:**

A *C1173T* polymorphism in intron 1 of the *VKORC1* gene has been claimed to determine the interindividual variability in the response to vitamin K antagonist therapy (VKA), but it is unknown whether it also influences bleeding risk. We aimed to confirm the relationship between *C1173T* status and phenprocoumon or acenocoumarol use, and to examine the risk of severe bleeding for the various genotypes.

**Methods and Findings:**

We studied this in a case-control study of 110 patients who bled during VKA therapy and 220 control patients free of bleeding under the same therapy. To achieve the same target INR, CT genotype and TT genotype control patients required less phenprocoumon (CC genotype 2.9 mg/d [95% confidence interval (CI): 2.6–3.2], CT genotype 2.6 mg/d [95% CI: 2.1–3.1], TT genotype 1.4 mg/d [95 % CI: 1.1–1.7]) or acenocoumarol (CC genotype 3.2 mg/d [95% CI: 2.9–3.5], CT genotype 2.3 mg/d [95% CI: 2.1–2.5], TT genotype 1.7 mg/d [95% CI: 1.3–2.1]) than CC genotype control patients. Compared with CC genotype individuals, carriers of at least one T allele had an increased risk of bleeding in the phenprocoumon users (crude odds ratio = 2.6, 95% CI: 1.2–5.7), but not in acenocoumarol users (crude odds ratio = 1.2, 95% CI: 0.6–2.3).

**Conclusion:**

These findings encourage taking further steps towards the evaluation of the use of *VKORC1* genetic testing for bleeding prevention in individuals who receive VKA therapy.

## Abstract 2

A polymorphism in the VKORC1 gene may affect the dose of vitamin K antagonists needed and the risk of bleeding after anticoagulation in some patients.

## Introduction

Vitamin K antagonists such as warfarin, acenocoumarol, and phenprocoumon are commonly used for the prevention and treatment of venous and arterial thrombosis. The molecular target of these anticoagulants is the vitamin K epoxide reductase complex of which the precise composition is largely unknown. Recently, one component of this complex, the vitamin K epoxide reductase complex subunit 1 (VKORC1), was identified by positional cloning [1]. This *VKORC1* gene is mutated in individuals with combined deficiency of vitamin K-dependent clotting factors type 2 or with warfarin resistance [1,2].

The required dose of antagonist in vitamin K antagonist therapy (VKA) to achieve a target level of anticoagulation is variable, in particular between individuals, but also within a single individual, and depends on, for example, dietary intake and variations in pharmacokinetics. Earlier this year, polymorphisms in the *VKORC1* gene have been reported that explain up to 30% of the variability in the pharmacological response to VKAs [3–5].

Bleeding is an important complication of treatment with VKA. In The Netherlands, intensive efforts by anticoagulation clinics are aimed at monitoring treatment with phenprocoumon and acenocoumarol, the two VKAs that are registered for use. These efforts are largely focussed on maintaining the level of anticoagulation, generally expressed as the international normalized ratio (INR), within a specified target range. In these efforts, individual patient characteristics such as those related to inherited determinants of the pharmacological response are only indirectly taken into account. We set out to estimate the contribution of a *C1173T* polymorphism in the *VKORC1* gene to the acenocoumarol and phenprocoumon dose requirement and to the bleeding risk.

## Methods

For the present analyses we used DNA from a previously reported case-control study [6]. This study includes 110 patients classified as “severe bleeders” and 220 “non-bleeders” (more than 96% of the participants are Caucasian) who were selected from two anticoagulation clinics. Major bleeding was defined as a bleeding that was clinically overt and met one of the following criteria: associated with a haemoglobin drop of ≥20 g/l, hospitalization, blood transfusion of two or more units, intracranial bleeding, intramuscular bleeding, intra-articular bleeding, or intraocular bleeding. Bleeders and non-bleeders were visited at home, during which a questionnaire was completed and blood was collected for the preparation of plasma and DNA. Genotyping for the *C1173T* polymorphism in the *VKORC1* gene was performed using a simple restriction enzyme digestion of PCR-amplified DNA. Details of the protocol are available from the corresponding author upon request. Statistical analyses were performed for acenocoumarol and phenprocoumon separately and combined. We calculated odds ratios as measures of relative risks from the contingency table as the exposure odds ratios (OR), with 95% confidence intervals (CI) based on the assumption of a Poisson distribution, according to Woolf [7]. The institutional review boards of the Academic Medical Center and the Leiden University Medical Center approved the study protocol, and all patients gave written informed consent.

## Results

Table 1 shows the dose requirements for the same target INR categorized for the different genotypes in controls. The data confirm the claims that were made in three earlier papers [3–5]. In comparison to CC genotype carriers, the average dose of acenocoumarol or phenprocoumon was 15%–30% less in CT heterozygotes, and about 40% less in homozygous TT individuals. There does not seem to be a major difference between acenocoumarol and phenprocoumon in this respect. The results are similar in the cases (data not shown).

**Table 1:**
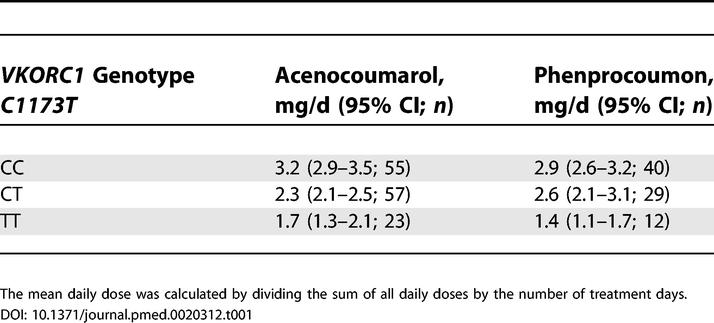
Relationship between *C1173T* Genotype and VKA Mean Daily Dose Requirement in Control Patients

Next we examined whether the *C1173T* genotype influenced the bleeding risk (Table 2). Overall the OR for major haemorrhage for carriers of at least one T-allele was 1.7 (95% CI: 1.1–2.5). When analysed separately, phenprocoumon seems to more strongly modify the bleeding risk of the *C1173T* genotype (OR = 2.6 [95% CI: 1.2–5.7 for T-allele carriers]), whereas in acenocoumarol users the genotype had little effect (OR = 1.2 [95% CI: 0.6–2.3]). When we add all controls together in the calculations of the odds ratios in order to obtain statistically more stable results (which is reasonable because genotype does not influence the prescription), the ORs for bleeding in T-carriers are 1.4 (95 % CI: 0.8–2.5) for acenocoumarol and 2.1 (95% CI: 1.1–4.2) for phenprocoumon.

**Table 2:**
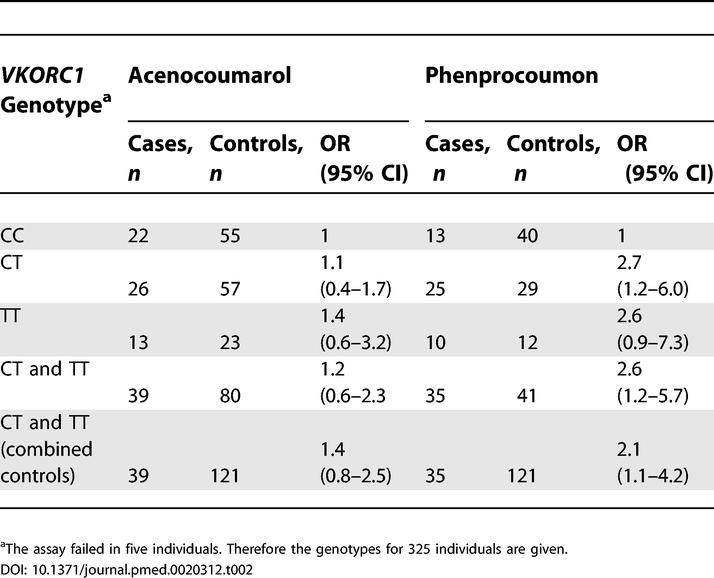
Numbers of Cases and Controls for the Different *VKORC1* Genotypes and Crude ORs for Bleeding under VKA Treatment

We examined the quality of anticoagulation in the various groups of genotypes and VKAs. The percentage of time in range was considerably higher in phenprocoumon treated controls (CC genotype 60% in range (95% CI: 45–75, CT/TT genotype 66% in range (95% CI: 62–71) than in individuals treated with acenocoumarol (CC genotype 41% in range (95% CI: 35–47, CT/TT genotype 46% in range (95% CI: 42–51), whereas genotype had no appreciable effect on time in range.

## Discussion

The results, although based on a small sample size of individuals with bleeding, support the suggestion that the bleeding risk for T-carriers is higher in phenprocoumon than in acenocoumarol users. If this finding is confirmed in additional studies and extended to the more frequently occurring and clinically relevant cases of nonmajor bleeding, it may imply that CT and TT carriers should be preferentially treated with acenocoumarol despite the fact that the genotype-related excess bleeding in phenprocoumon users is not mediated through unstable anticoagulation that is detected by the INR, because genotypes were associated with bleeding but not with unstable INR values. In fact, stability of treatment is better in phenprocoumon than in acenocoumarol users, which is in agreement with previous publications [8]. This paradox—apparently good INR control by all measures, but higher bleeding risks in phenprocoumon-treated patients—has been reported before, in particular in relationship to minor bleeding [8].

At present we have no clear explanation for risk differences between the two coumarin anticoagulants. It is unlikely that these drugs have differential effects on the carboxylation status of vitamin K–dependent clotting factors. In support of this, levels of factors IX (which do not influence the INR) are similar between the two treatment groups (data not shown). More likely, a difference in bleeding risk may find an explanation in the vastly different pharmokinetics of acenocoumarol (biological half-life = 11 h) and phenprocoumon (half-life = 140 h). Receivers of anticoagulants are often frail people with significant health problems. Perhaps alterations in their health status that affect coumarin sensitivity are not effectively managed with a long half-life product.

The increased bleeding risks give support to the notion that genotyping for this polymorphism in the *VKORC1* gene may go beyond an academic interest and may become standard for every individual starting on acenocoumarol and phenprocoumon treatment. It may help in the identification of those individuals who are at the highest bleeding risk and thus should be monitored more intensely. Such *VKORC1* testing may need to be complemented with testing for *Cyp2C9* polymorphisms, which also influence the pharmacokinetics of VKAs and for which there is also increasing evidence for a relevant relationship between genotype and bleeding [9].

**Patient Summary**

**Background**

Patients who have experienced blood clots or who are at risk of getting clots are often put on anticoagulant treatment to reduce the ability of blood to clot efficiently. One group of these drugs works by interfering with the action of vitamin K, which is essential for blood clotting. The disadvantage of this type of drug is that the treatment has to be monitored closely to ensure that it does not work too well, in which case a patient may have bleeding, or not well enough, in which case the blood may clot.

**Why Was This Study Done?**

Previous work has suggested that a change in one of the genes involved in blood clotting, *VKORCI*, might affect how well these drugs work. The authors wanted to confirm this finding and see if changes in the gene also affect how likely patients are to bleed when given the drugs.

**What Did the Researchers Do and Find?**

They looked at 110 people who had bled after being given the anticoagulants and 220 who had not. They found that one particular variation in the gene resulted in people requiring less anticoagulation medicine. This same variation also meant that some people were more likely to bleed with one of the anticoagulant drugs, but not with another.

**What Do These Findings Mean?**

If these findings are confirmed in studies of other groups of patients, they suggest a reason why patients vary so much in their response to anticoagulation and why some people bleed whereas others do not. In the future, it may be possible to look at the genes in patients given anticoagulation to help make decisions about their treatment.

**Where Can I Get More Information Online?**

MedlinePlus has information on blood clots and the drugs used to treat them:

http://www.nlm.nih.gov/medlineplus/bleedingdisorders.html

Anticoagulation Europe is a site specifically for people taking anticoagulants:

http://www.anticoagulationeurope.org/

## Abbreviations

CI: confidence interval
INR: international normalized ratio
OR: odds ratio
VKA: vitamin K antagonist therapy
